# Microstructured Waveguides with Polyelectrolyte-Stabilized Gold Nanostars for SERS Sensing of Dissolved Analytes

**DOI:** 10.3390/ma11050734

**Published:** 2018-05-05

**Authors:** Daniil N. Bratashov, Natalia A. Burmistrova, Sergey D. Bondarenko, Boris N. Khlebtsov, Vsevolod S. Atkin, Andrey A. Shuvalov, Anastasiya A. Zanishevskaya, Yulia S. Skibina, Irina Y. Goryacheva

**Affiliations:** 1 Remote Controlled Theranostic Systems Lab, Saratov State University, Astrakhanskaya 83, 410012 Saratov, Russia; khlebtsov_b@ibppm.ru (B.N.K.); ceba91@list.ru (V.S.A.); 2 Institute of Chemistry, Saratov State University, Astrakhanskaya 83, 410012 Saratov, Russia; naburmistrova@mail.ru (N.A.B.); bondsd90@gmail.com (S.D.B.); shyller8905@gmail.com (A.A.S.); zan-anastasiya@yandex.ru (A.A.Z.); goryachevaiy@mail.ru (I.Y.G.); 3 Institute of Biochemistry and Physiology of Plants and Microorganisms, Russian Academy of Sciences, Prospekt Entuziastov13, 410049 Saratov, Russia; 4 SPС Nanostructured Glass Technology Ltd., 50 years of October av. 101, 410033 Saratov, Russia; skibinajs@yandex.ru

**Keywords:** microstructures waveguide, SERS, fiber-enhanced Raman scattering

## Abstract

A sensor based on microstructured waveguides (MWGs) with a hollow core inner surface covered with polyelectrolyte-layer-stabilized gold nanostars was developed for the SERS sensing of dissolved analytes. A polyelectrolyte-layer coating over the inner surface of glass cladding served as a spacer, reducing nonlinear optical effects in the glass near plasmonic hotspots of nanoparticles, as a stabilizing agent for thermodynamically unstable gold nanostars and as an optical coating for the fine-tuning of MWG bandgaps. This approach can be used to construct different kinds of SERS sensors for dissolved analytes, providing conservation, the prevention of coagulation, and the drying of a liquid sample for the time required to record the signal.

## 1. Introduction

A photonic crystal fiber (PCF) and microstructured waveguide (MWG) are the light-waveguided devices that consist of a central core and a periodic grating structure of claddings formed by either welded microcapillaries or holes in the solid structure [1,2,3,4,5]. There can be a number of different fiber structures (PCFs) that have light-waveguiding properties [6]. The two major types of PCFs are solid core PCFs (SC PCFs) and hollow core PCFs (HC PCFs), containing high and low refractive index material in the core, respectively. PCF waveguiding properties can be enabled by two modes of work: an effective refractive index contrast between core and claddings providing total internal reflection such as conventional fibers, and a photonic bandgap mode in which light can be confined in a lower refractive index core or hollow core. Inside the PCF, all nonlinear optical effects are greatly enhanced, which has many practical applications, the most important one of which is supercontinuum generation from short laser pulses [7]. Another possible nonlinear effect in PCFs is Raman scattering enhancement.

Raman spectroscopy is an advanced label-free analytical method that can be used for the monitoring of chemical reactions in situ, the measurement of analytes in their natural form, the ability to measure gases and samples dissolved in liquid, and so on [8,9,10]. The main problem of Raman analytics—its inherent low sensitivity due to small scattering cross sections for most of the actual analytes of interest—is now more or less solved by surface-enhanced Raman scattering (SERS). An amplification of Raman scattering signals of the molecule is either due to an enormous electromagnetic field enhancement near the surface with active plasmon modes or due to charge transfer to the analyte molecule from light adsorbing material. SERS has a strong detection potential (up to single molecule), but the major problem now is the low reproducibility of enhancement required for quantitative measurements. There is always the dilemma of high enhancement factor of SERS versus high stability. High stability of signal enhancement can be provided by a number of methods such as precise manufacturing of photonic devices [11,12], controlled overgrowth of gold leaflets [13], or spacer-controlled structures [14].

Measuring of Raman and SERS signals from analytes in liquid in a predictable manner is not so simple when using micro Raman equipment. The small liquid droplet is drying rapidly, the liquid can be volatile, and liquid samples such as fresh blood can coagulate, so the concentration of the analyte can change rapidly during measurement. The focus of the objective lens and the numerical aperture of light collected by it inside the liquid can also change rapidly during measurement due to drying. This complicates quantitative measurements of Raman signals. There are also problems of sample nonuniformity and of effects such as the “coffee ring” that make quantitative Raman analytics even more problematic. The problem can be solved by fiber Raman spectroscopy in cuvettes, but this requires a large amount of samples that can be hard to obtain (especially in biology experiments).

Fiber-enhanced Raman scattering and its combination with the SERS effect is a promising approach in providing quantitative, reproducible Raman analytics. It keeps the analyte in the dissolved form, protecting it from drying, evaporation, coagulation, and other sample degradation processes, also keeping initial analyte concentration during long periods of time. It integrates the signal along the fiber length, thus providing so-called fiber-enhanced Raman scattering [15] and amplifying it with SERS [16] if necessary, thus solving the problem of point-to-point and measurement-to-measurement reproducibility required by quantitative Raman analytics. It solves the problems of nonuniform SERS enhancement factor distribution along the sample, of nonuniform analyte concentration, and of providing constant focusing and constant numerical aperture of the optical signal registration system [17]. It allows for the measurement of the ultralow concentration of analytes in the very low sample volumes. On the other hand, there are new problems that arise in fiber-enhanced Raman scattering, and they involve proper waveguiding, bandgap positions, and amplification of the backgrounds in the fiber.

Fiber-enhanced Raman spectroscopy has already been used for measuring the concentration of antibiotics such as moxifloxacin [18] or levofloxacin [19] in sub-µL or nL volumes. It can also be combined with the SERS signal enhancement, increasing the sensitivity of such platforms. Fiber enhancement combined with the SERS effect has been used to detect Rhodamine 6G [20,21], Rhodamine B [22], benzenethiol [23] dissolved in ethanol, and different analytes in red blood cells such as bilirubin and biliverdin [24]. The SERS effect can be achieved by coating the internal surface of the fiber core with plasmonic nanoparticles or a rough metal surface [25], but such coating does not cause additional problems only with fused silica fibers. There is a problem of fiber backgrounds while measuring Raman signals inside PCFs [26]. If a PCF is made from glass, all nonlinear effects are significantly enhanced in the high electromagnetic field near the plasmonic hotspots inside the fiber, making the background signal from glass claddings very high. The internal surface of glass fiber can be modified by polyelectrolyte adsorption [27] making the spacer between the hotspots on the metal surface and the glass fiber claddings. This approach allows for the use of relatively cheap low fluorescence glass instead of fused silica for production of PCFs and provides some charge selectivity of the platform.

The ends of fibers should provide effective coupling of light with external optics, so they should be cleaved. If open-end fibers are used, liquid can penetrate the cladding holes, which is in many cases undesirable. This problem is usually solved by sealing the fiber ends either by adhesive polymers, photocurable composites, or precise welding by heating. The sealed part of the fiber has altered light propagation properties, so it should be as short as possible. This can be done for example by cleaving fiber ends after their sealing as close as possible to the unaltered fiber structure. Another possible approach is to work with the surface tension, making small holes hydrophobic and meniscus too small to allow liquid to flow into small capillaries, allowing the liquid to only flow into the central core. The excitation laser wavelength and Stokes Raman signal range should be in the transmission window of the PCF after all internal structure layers are formed and the core is filled with a liquid containing the analytes of interest [28].

The goal of our work is to provide a reliable and stable sensor structure inside the MWG made from soft glass with a SERS active layer that consists of gold nanostars inside the layer-by-layer assembled polyelectrolyte coating. A polyelectrolyte-layer coating over the inner surface of glass cladding serves as a spacer to reduce nonlinear optical effects in the glass and thus reduce the intense fluorescence background, as a stabilizing agent for thermodynamically unstable gold nanostars, and as an optical coating for fine-tuning of MWG bandgaps.

## 2. Materials and Methods

MWG samples with different concentric ring cladding geometries were formed from the soft glass as described before [29].

Poly(sodium 4-styrenesulfonate) (PSS, 70 kDa), poly(allylamine hydrochloride) (PAH, 15 kDa), polyvinylpyridine (PVPyr, 160 kDa), and poly(ethyleneimine) (~50% H_2_O, PEI, 600–1000 kDa) polyelectrolytes and Rhodamine B solutions were purchased from Sigma-Aldrich (St. Louis, MO, USA). Gold nanostars were obtained by the protocol described earlier [30] using 10 nm Au seeds. HAuCl_4_, HCl, AgNO_3_, and ascorbic acid used in this part were purchased from Sigma-Aldrich. All chemicals were used as received without further purification. Deionized water (specific resistivity higher than 18.2 MΩ∙cm) from Milli-Q plus 185 (Millipore, Merck KGaA, Darmstadt, Germany) water purification system was used to prepare all solutions.

The modification of the inner surface of the MWG was performed layer by layer. It included (i) pretreatment with a cleaning solution (H_2_SO_4_:H_2_O_2_ = 3:1, 30 min), (ii) successive incubation with polymer solutions (2 min), and (iii) incubation with gold nanostars (1 min). MWG samples were rinsed with distilled water and dried under an argon stream after each step.

SERS and Raman measurements were taken using Renishaw inVia microscope and a 785 nm near-IR laser at 3 mW of power. Signals for hollow-core MWG were obtained with a 5×/0.12 n.a. Leica nPlan objective lens for coupling with MWG in a backscattering configuration. Backscattered Rayleigh light was used to determine the optimal position of MWG relative to the objective lens.

**Figure 1:**
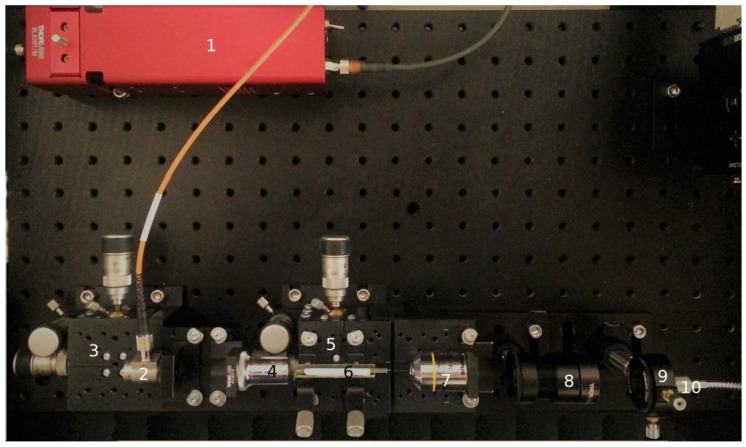
Optical setup for measuring UV-vis-NIR transmission of photonic crystal fibers (PCFs). **1**—whitelight source, **2**—whitelight source fiber collimator, **3,5**—XYZ manual stages, **4**—40× objective lens, **6**—PCF in the cuvette, **7**—10× objective lens, **8**—collimator lens, **9**—XY manual fiber position adjustment, **10**—fiber spectrometer entry fiber.

Optical transmission of MWG in UV-vis-NIR range was measured with the self-made setup shown in Figure 1. White light from Thorlabs SLS201/M stabilized tungsten–halogen lamp source (360–2600 nm, 10 mW optical power, ThorLabs Inc., Newton, NJ, USA) with output multimode fiber was collimated into a parallel beam by an RC02FC-P01 mirror collimator. The collimated incident light was coupled into MWG by a 40×/0.65 NA objective lens (LOMO, St Petersburg, Russia). MWG and incident light optics can be moved precisely by 3D micropositioning stages MBT610D/M (ThorLabs Inc., Newton, NJ, USA). Light from the MWG was collected by a 10×/0.25 NA objective lens (Olympus, Shinjuku, Tokyo, Japan), collimated into spectrometer entrance fiber by an achromatic doublet lens (ThorLabs Inc., Newton, NJ, USA) and measured by an Avantes AvaSpec-HS2048XL (Avantes BV, Apeldoorn, the Netherlands) fiber-optics spectrometer.

SEM measurements of MWGs were taken with a Tescan MIRA II LMU (Tescan, Brno, Czech Republic). Short fragments of MWGs were cleaved perpendicularly with a ruby fiber cleaver and fixed with conductive tape. Before imaging, the samples were coated with an approximately 5 nm thick gold film using an Emitech K350 sputter-coater (Quorum Technologies Ltd, Ashford, UK). The images were taken at a 30 kV accelerating voltage.

## 3. Results and Discussion

**Figure 2:**
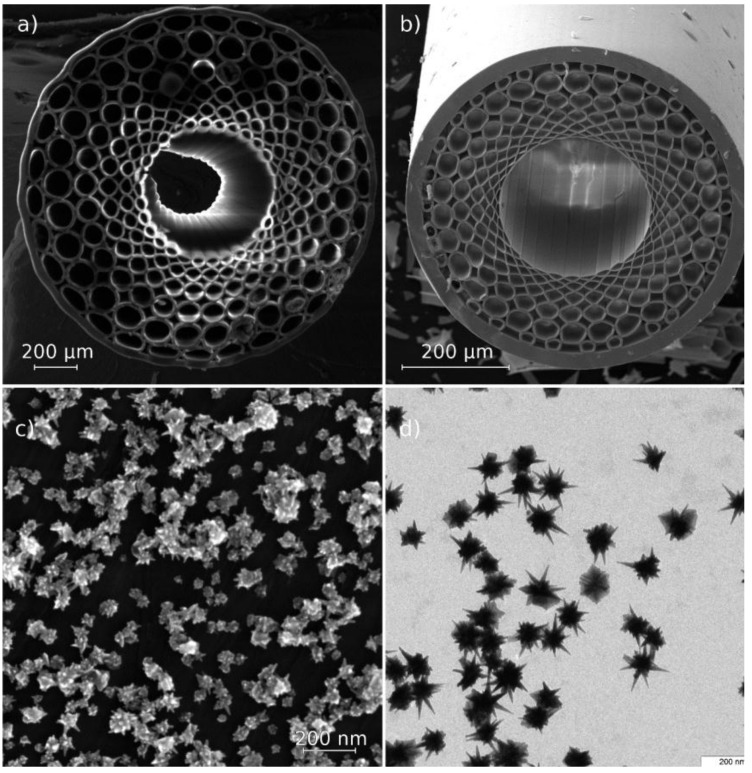
SEM image of constructed HC MWG structures made from soft glass (**a**) and low-fluorescence glass (**b**), SEM image of gold nanostars coating (**c**), and TEM image of its dried suspension (**d**).

To make a SERS substrate from HC MWGs, we chose a 785 nm laser excitation wavelength and constructed the structure of MWGs such that there were no photonic bandgaps on this wavelength or in the Stokes-shifted Raman fingerprints region. The SEM image of constructed fiber structures is shown in Figure 2. The internal glass layer around the hollow core (the fiber geometry is shown in Figure 2b) was made of low fluorescence glass, reducing nonlinear optical effects that show a high background signal in Raman measurements.

To make the SERS substrate, HC MWG claddings were closed by welding. A composite coating containing both polyelectrolyte layers and plasmonic gold nanostars was then adsorbed on the inner surface of the MWG core. In our first experiments, we tested coatings for the self-assembly SERS substrates on glass such as PVPyr [31] and PEI, but there were too many nonlinear optical signals from the glass claddings amplified by plasmonic nanoparticles inside the MWG. Therefore, a spacer between the glass and gold nanostars that consisted of highly polar polyelectrolyte molecules with low Raman cross sections was provided. We applied the technique of layer-by-layer coating on the outer wall of the hollow MWG core [27]. By measuring the signal from SERS substrates with a different layer structure, we found that four bilayers of PAH/PSS over the initial PEI layer adsorbed on the glass surface provided enough distance to make the glass signal negligible. As the final layer of all structures, we used gold nanostars [30], one of the most effective single-particle enhancers of Raman signals. A TEM image of the nanostars is shown in Figure 2d, and an SEM image of the stars in the coating is shown in Figure 2c. However, the nanostars themselves were not thermodynamically stable nanostructures. They were susceptible to aggregation and Ostwald ripening processes; therefore, initially, an effective SERS platform degrades rapidly in solution and can only be used for a short time after it is made (i.e., a few hours before aggregation; in 1–2 days, it can be refreshed by intense ultrasound agitation). Therefore, it is difficult to choose the right stabilizer coating with a low thickness that renders the particles stable, that allows analytes to flow into hotspots on nanostar edges, and that has itself very low Raman cross section, such that the signal of the analyte of interest will not be altered by the coating. The gold nanostars sunk into polyelectrolyte layers, thus providing a stable SERS platform during several weeks of testing. After all, coatings were adsorbed, and the welded glass claddings were reopened by cleaving the fiber to obtain good optical coupling in further experiments. Therefore, the final structure of composite coating consisted of hollow core glass PCF/PEI/(PAH/PSS)_4_/gold nanostars.

**Figure 3:**
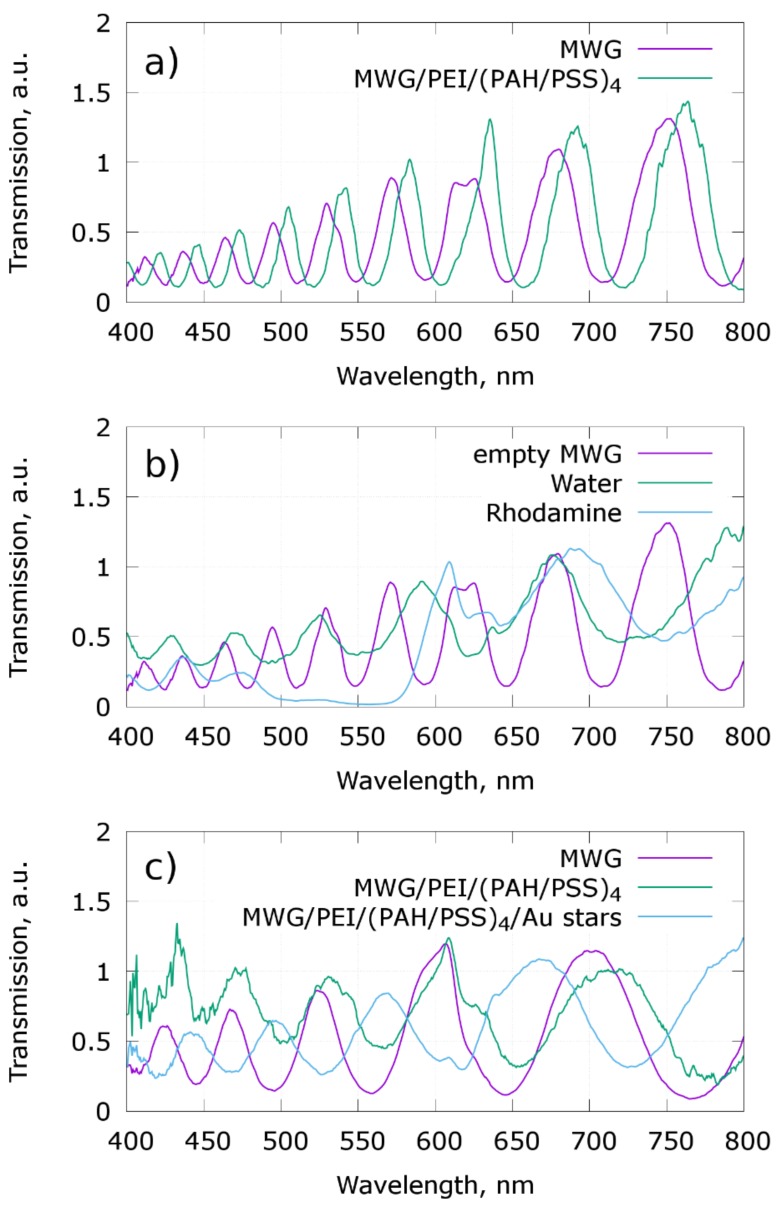
Transmission of HC MWGs after adsorption of polyelectrolyte layers (**a**), after filling it with water and Rhodamine B (**b**), and after making the SERS substrate on low fluorescence glass fiber (**c**).

The main problem of SERS sensor construction inside PCFs is the high sensitivity of photonic bandgaps to core and cladding hole refractive indexes. For effective Raman detection, the wavelengths of incident laser light and the fingerprint regions of Raman spectra should be in the light-propagating spectral band of the PCF. Making plasmonic active layers and intermediate layers inside the PCF, filling the central core and/or claddings or channel inside the claddings by the liquid analyte, and the evaporation of the solvent are all process that change the bandgaps of PCFs drastically (Figure 3). The effects of bandgaps also depend on an effective refractive index contrast, so the adsorption of polyelectrolytes on the soft glass capillary changes it slightly (Figure 3a), but if the low fluorescence glass with a slightly altered refractive index is used, the bandgaps are changed drastically, moving bandgaps into the centers of the former light propagation modes (Figure 3c). All of the above-mentioned effects are even more pronounced if the cladding voids are also filled with liquid (Figure 3b), and it can even make the PCF non-waveguiding if the refractive index of the liquid is too close to the refractive index of the glass. There is also a low transmission region, 500–570 nm, on the transmission spectrum of MWGs filled with a Rhodamine B water solution (Figure 3b) due to the Rhodamine B adsorption of light in this wavelength range.

**Figure 4:**
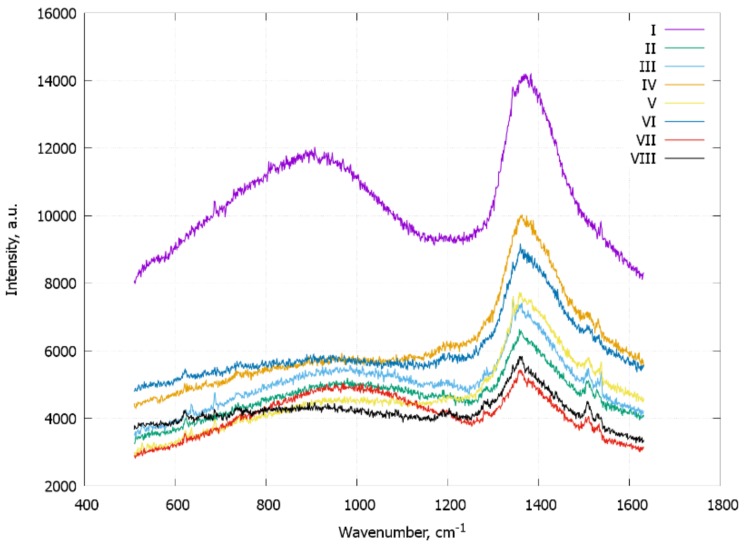
The enhanced fluorescence signal of MWGs dependence on a number of polyelectrolyte coating layers. The typical spectra are shown. The signal varies from fiber to fiber.

Another large problem using soft glass MWGs for SERS sensing is the high fluorescent background with intensities much higher than those of the Raman signal. We have tried to reduce it by adding layer-by-layer polyelectrolyte coatings with small Raman cross sections between the plasmonic particles and the glass of the internal surface of MWG cores. Each bilayer of PAH/PSS polyelectrolytes add 1.4 nm of the overall coating thickness [32], but the layer is not solid, and it is penetrable by small particles or its sharp edges. In general, high electrical field enhancement near the plasmonic hotspots extends in the single-nm range that corresponds to the single bilayer. In our experiments with the high fluorescence glass MWGs, the initial high intensity of fluorescence was reduced even by a single layer of polyelectrolytes, but the background becomes stable (not changing from fiber to fiber) on the fourth bilayer adsorption (Figure 4).

**Figure 5:**
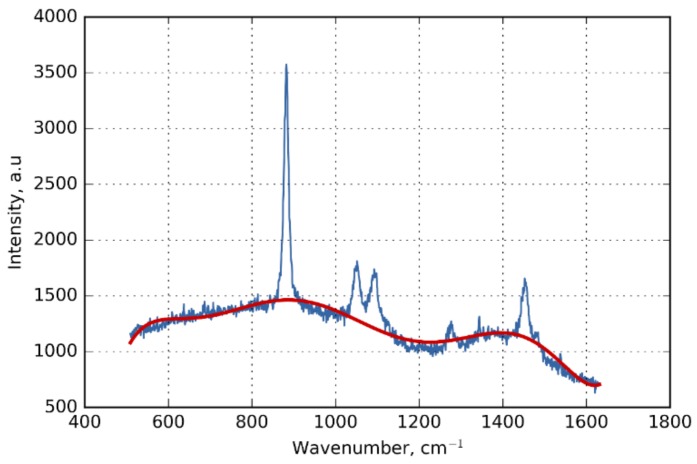
Fiber-enhanced SERS spectrum of ethanol inside the liquid-filled core of the MWG. The approximated polynomial background is shown by a red line.

The obtained MWG SERS sensors were tested by filling it with ethanol and measuring the Raman signal. The SERS signal was measured by coupling a short 10 mm trunk of MWGs with a microspectrometer through a 5×/0.12 n.a. objective lens in a backscattering configuration. Raman signal was measured with a 785 nm laser excitation wavelength, 30 mW of laser power, and a 10 s integration time. The signal is shown in Figure 5. The SERS signal is much stronger than the background of soft glass that is not removed on the graph and shown separately by the red line.

**Figure 6:**
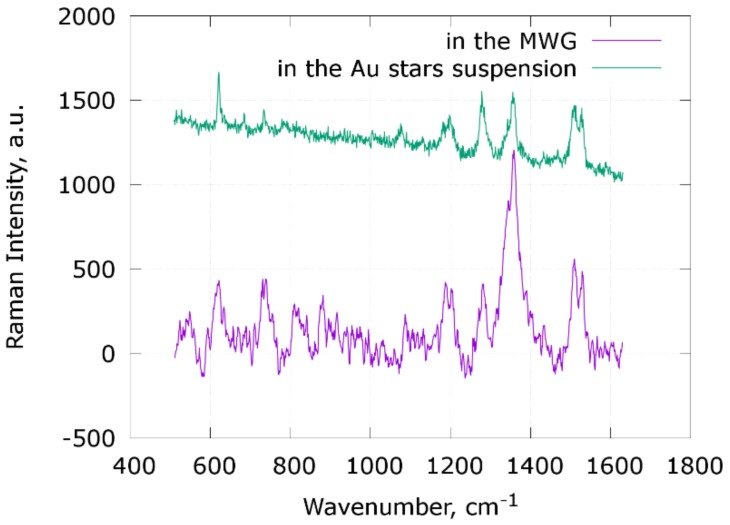
SERS spectra of a 1 mM Rhodamine B solution inside the low fluorescence HC MWG with PEI(PAH/PSS)_4_/Au stars coating and Rhodamine B mixed 1:1 with the same Au nanostars.

The sensing ability of the constructed MWG SERS sensors was tested with a 1 mM Rhodamine B water solution. The results are shown in Figure 5. For reference, the same solution was measured by directly mixing it 1:1 with the freshly made gold nanostar suspension. The SERS measurements of the Rhodamine B suspension was made using a 50×/0.5 n.a. objective lens, laser excitation with a wavelength of 785 nm, 3 mW of laser power, and a 10 s signal integration time and shown in Figure 6 as a reference spectrum. The SERS signal of Rhodamine B inside the HC PCF was measured by coupling it with spectrometer through a 5× objective lens using a 785 nm laser wavelength, 3 mW of power, and 60 s of integration time. The signal intensities corrected to integration time are comparable in this case; however, intensities of individual Raman lines are different due to different attenuation of the signal by the fiber bandgap’s structure. Nevertheless, the Rhodamine B spectrum is detectable, all the lines can be identified, and the spectrum itself can be found in the reference spectra database. It is worth noting that the suspension of unstabilized gold nanostars rapidly degrades in only a few hours, as it is thermodynamically unstable and susceptible to Ostwald ripening and aggregation processing, rapidly losing SERS sensitivity. SERS measurements inside the MWG were made with delays varying from 1 day to a month, and measured signals were similar, so the stars in the polymer layer of the sensor are shown to become thermodynamically stable.

## 4. Conclusions

SERS sensors based on soft glass HC MWGs with optimized cladding structures, polyelectrolyte coatings of the internal glass surface of the inner wall of the claddings, and plasmonic-active gold nanostars were constructed. To reduce glass cladding fluorescence enhancement by plasmonic gold nanostars, the polyelectrolyte-based spacer with low Raman cross sections was constructed between the gold particles and the glass wall of MWGs using the layer-by-layer technique. The effects of the layer-by-layer polyelectrolyte coating, plasmonic gold nanoparticle adsorption, and the filling of a hollow core with the analyte solution on the photonic bandgap structure of the fiber were investigated. The proper structures of soft glass MWGs taking into account these effects were elaborated. The SERS sensors were tested with ethanol and a 1 mM water solution of Rhodamine B. Gold nanostars inside the sensor were stabilized by polyelectrolyte coating, but still maintained SERS enhancement at the same level as in the solution. Such construction of MWG-based SERS sensors allows for the measurement of analytes in dissolved or liquid form with long acquisition times and for the protection of the sample from drying, evaporating, and coagulating during measurements.
